# The attributes of plakins in cancer and disease: perspectives on ovarian cancer progression, chemoresistance and recurrence

**DOI:** 10.1186/s12964-021-00726-x

**Published:** 2021-05-17

**Authors:** Tamsin Wesley, Stuart Berzins, George Kannourakis, Nuzhat Ahmed

**Affiliations:** 1 Fiona Elsey Cancer Research Institute, Ballarat Technology Central Park, Suites 23-26, 106-110 Lydiard Street South, Ballarat, VIC 3353 Australia; 2 School of Science, Psychology and Sport, Federation University Australia, Ballarat, VIC 3010 Australia; 3 Department of Obstetrics and Gynaecology, University of Melbourne, Melbourne, VIC 3052 Australia; 4 Centre for Reproductive Health, The Hudson Institute of Medical Research and Department of Translational Medicine, Monash University, Melbourne, VIC 3168 Australia

**Keywords:** Plakins, Ovarian cancer, Tumour cells, Ascites, Chemoresistance, Chemotherapy

## Abstract

The plakin family of cytoskeletal proteins play an important role in cancer progression yet are under-studied in cancer, especially ovarian cancer. These large cytoskeletal proteins have primary roles in the maintenance of cytoskeletal integrity but are also associated with scaffolds of intermediate filaments and hemidesmosomal adhesion complexes mediating signalling pathways that regulate cellular growth, migration, invasion and differentiation as well as stress response. Abnormalities of plakins, and the closely related spectraplakins, result in diseases of the skin, striated muscle and nervous tissue. Their prevalence in epithelial cells suggests that plakins may play a role in epithelial ovarian cancer progression and recurrence. In this review article, we explore the roles of plakins, particularly plectin, periplakin and envoplakin in disease-states and cancers with emphasis on ovarian cancer. We discuss the potential role the plakin family of proteins play in regulating cancer cell growth, survival, migration, invasion and drug resistance. We highlight potential relationships between plakins, epithelial-mesenchymal transition (EMT) and cancer stem cells (CSCs) and discuss how interaction of these processes may affect ovarian cancer progression, chemoresistance and ultimately recurrence. We propose that molecular changes in the expression of plakins leads to the transition of benign ovarian tumours to carcinomas, as well as floating cellular aggregates (commonly known as spheroids) in the ascites microenvironment, which may contribute to the sustenance and progression of the disease. In this review, attempts have been made to understand the crucial changes in plakin expression in relation to progression and recurrence of ovarian cancer.

**Video Abstract**

**Supplementary Information:**

The online version contains supplementary material available at 10.1186/s12964-021-00726-x.

## Background

The plakins are a large versatile family of proteins present in different tissues of the body that are well known for their roles in providing cytoskeletal integrity and organizational support to cellular adhesion complexes [1]. They provide strength to cells exposed to mechanical stress, such as muscle and skin, linking intermediate filaments that form the cell cytoskeleton and mediate cadherin associated cell–cell junctions to provide tissue integrity [1, 2]. Plakins also connect hemidesmosome junction complexes to the plasma membrane, nucleus and mitochondria of human cells and play a crucial role in maintaining cytoskeletal stability while at the same time act as adaptors for signalling proteins that regulate cell-extracellular matrix connections, cell–cell connection, cell migration and invasion, differentiation, and in some cases stress responses. The participation of plakins in intracellular signalling, cellular migration and differentiation makes this family of proteins an intriguing subject for cancer research [3].

Mammalian plakins are evolutionarily conserved and have a similar cellular organization in different tissues [2]. However, they have multiple binding sites and isomeric variations that provide them with additional roles across a range of tissues [2]. Their varied composition and binding patterns with hemidesmosomes and intermediate filaments affect tissue integrity in genetic and autoimmune diseases [2]. The most known plakins are plectin (PLEC) and desmoplakin (DSP). The remainder are envoplakin (EVPL), periplakin (PPL) and Epiplakin (EPPK1). Their cousins are the spectraplakins, microtubule-actin cross-linking factor (MACF1 also known as ACF7) and bullous pemphigus antigen 1 (BPAG1). Often the epithelial and neuronal isoforms, BPAG1e and BPAG1n are grouped with the plakins, while BPAG1a and 1b are grouped with the spectraplakins, the division being based on their similar characteristics to spectrin family proteins [2].

Most of our current knowledge on the role of plakins in humans comes from studies of mammalian tissues such as skin and skeletal muscles [1]. However, very little is known about how the assembly of plakins that incorporates intermediate filaments and adaptor proteins changes with cellular transformation associated with neoplastic transformation. As a result, the molecular mechanisms that maintains plakin assembly with other adaptor and scaffolding proteins to provide cytoskeletal stability in cancer cells remains vague. In this review, we summarize our knowledge of plakins in skin and skeletal muscle biology, give an overview of recent findings about plakin biology in cancer, and discuss these findings in the setting of ovarian cancer progression and recurrence.

## Structure of common plakins

**Fig. 1:**
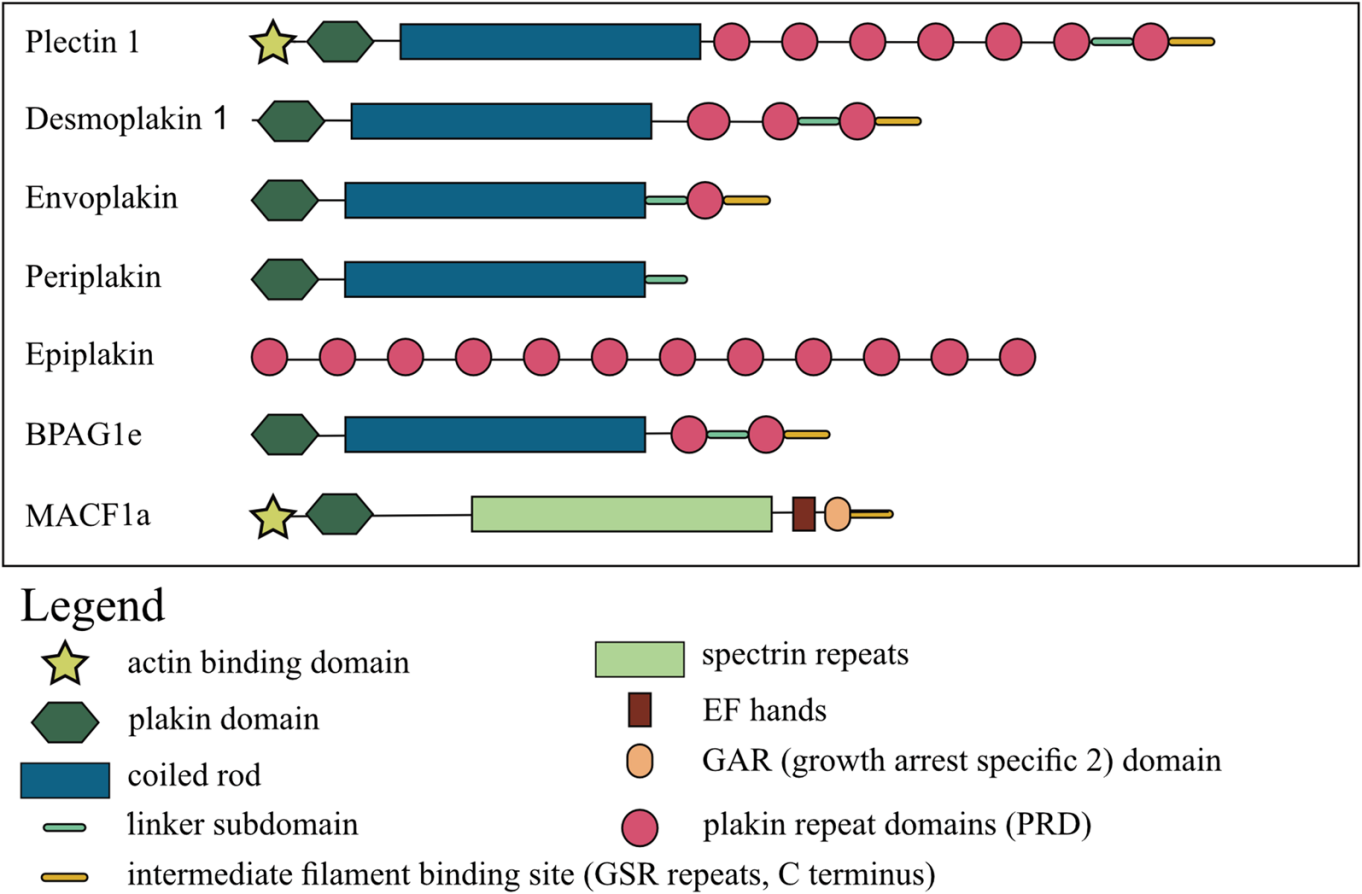
Examples of plakin and spectraplakin structure, not to scale (T Wesley unpublished) [2, 11, 48, 180]

Plakins are large multidomain versatile proteins that the shape the cytoskeleton of cells by linking to different microfilaments, intermediate filaments or microtubules [4]. They also connect different cytoskeletal networks within the cells and are also involved with linking the cytoskeletal networks to different sites on the plasma membrane, nuclear membranes or different organelles within various tissues [2]. All conventional plakins share a common structural design which comprises of a NH2-terminal head region (plakin domain), a central coiled rod domain and a COOH-terminal tail domain [5]. The plakin domain dominates the head region of these proteins, which is shared by mammalian plakin members [3]. In the case of EVPL, PPL and DSP the head domain also consists of a number of spectrin repeats and a Src homology 3 domain (SH3) [3]. The N-terminal end of plakins enables protein–protein interactions, for example, PPL, which interacts with PLEC and kazrin [6, 7], while DSP at the N termini interacts with plakoglobin, also known as γ-catenin [8]. The plakin domain in the N-terminal end also connects cell adhesion complexes and cytoskeletal networks essential for sustaining cellular architecture and maintaining tissue integrity and stability under stress conditions [3, 9, 10]. The central coiled region is involved with protein–protein dimerization, which provides strength to cytoskeleton and cell junctions, while the C-terminal region consists of a few plakin repeat domains that interact with intermediate filaments [3, 11] The structural components of the plakins are illustrated in Fig. 1.

## Common plakin members: their structure and function in normal cell biology

### Desmoplakin (DSP)

DSP has two isotypes. The first (DSPI) is about 330 kDa in size and is predominantly found in cardiac muscle whereas the second (DSPII) is about 250–260 kDa, with a shorter rod domain than DSPI. The N terminal domain of DSP is where the interaction with the desmosomal proteins, including cadherins and armadillo proteins occurs. The C-terminus connects to intermediate filaments such as desmin, vimentin and keratins [8, 12, 13].

DSP is the major protein involved with desmosome formation and cell–cell adhesion [10, 14]. It has been established that DSP can be degraded by the 26 s proteasome, thus modulating DSP expression and signalling [15]. The protein p53, known for its control of the cell cycle and apoptosis, and the related p63 are involved in the regulation of the structure and function of desmosomes. Both p53 and p63 regulate the expression of many desmosomal proteins, including DSP [16]. The tumour suppressor function of the p53 family also modulates DSP expression [16].

### Envoplakin (EVPL)

EVPL is often studied in association with PPL. Both proteins were discovered through study of the skin-blistering disease, paraneoplastic pemphigus (PNP), which accompanies both benign and malignant neoplasia, diagnosed by the production of autoantibodies against proteins in the cells of skin and mucus membranes that breaks cell–cell communication resulting in skin erosion and blistering [3]. In addition, EVPL is found in a range of epithelial cells of various tissues and links intermediate filaments (IFs) and desmosomes to the cornified envelope of mature cells of stratified squamous epithelium [4]. In skin cells that have not yet formed a cornified envelope, EVPL and PPL are found near DSP at desmosomes, in an inter-desmosomal network at the cell membrane associated with keratin filaments [4, 5]. EVPL has a p63 specific response element and its expression in the skin of mice was shown to be severely reduced in the absence of p63 protein [17]. EVPL is predicted to form heterodimers with PPL due to its rod domain sequence. The binding sites for intermediate filaments, including vimentin, are found in its single plakin repeat domain [5, 18].

### Periplakin (PPL)

PPL is a 195 kDa protein with similar structure and function as EVPL. It acts as a cytolinker between intermediate filaments and cytoskeletal proteins and forms an integral part of desmosomal plaques with cadherins and other members of the plakin family such as DSP, PLEC and EVPL [19]. In addition, PPL interacts with other non-desmosomal proteins at the plasma membrane to regulate the signalling pathways including, AKT [20], Annexin 9 [21], CD64 [22] and melanin concentrating hormone receptor 1 (MCHR1) [23]. PPL’s intermediate filament binding domain is at its COOH terminus end, where it specifically binds vimentin and keratin 8 [5, 24]. Similar to other plakins, both PPL and EVPL associate with the plasma membrane through the NH2 terminus end [5]. In the cornified envelope of stratified squamous epithelial cells PPL crosslinks intermediate filaments and desmosomes and forms a heterodimer with EVPL [4, 24].

PPL is found in a wider range of human tissues, especially in tissues that are continuously exposed to mechanical dynamics such as heart, skeletal muscle and lungs. It is strongly expressed in both proximal and distal airway epithelium of lungs [25], and a recent study has shown that PPL expression is downregulated in the lung especially in the alveolar epithelial cells as a result of bleomycin-induced injury in a mouse model [26]. In the same study it was shown that deletion of PPL gene in mice improved survival from bleomycin-induced lung injury due to enhanced expression of anti-inflammatory cytokines, reduced expression of pro-fibrotic mediators and diminished response to TGFβ signalling [26], suggesting that the expression of PPL may have a role in initiating a anti-inflammatory response in lungs.

### Epiplakin (EPPK1)

EPPK1 is a 550–700 kDa protein [27] and is found as a single chain structure due to its lack of a dimerization motif. It has been identified in the oesophagus and mucous epithelial cells of the colon and stomach and is present in the epidermis and glandular cells of parotid and sweat glands [28]. In the epidermis, EPPK1 co-localises with tight junctions (zonulae occludens or occluding junctions), specifically with ZO-1, a tight junction marker. ZO-1 is an intracytoplasmic protein that binds to junction adhesion molecules, occludin and claudin. EPPK1 is mostly concentrated in the upper layers of the epidermis, but not the granular layer whereas tight junctions are found in both the granular and upper layers. The resistance of the upper layers to shrinkage is assisted by EPPK1 [29].

EPPK1 binds to intermediate filaments, particularly keratin 8 and 18. It may be involved in intermediate filament phosphorylation, or binding of focal adhesion molecules, such as tyrosine-protein kinase Src, to intermediate filaments [29]. During wound healing, EPPK1 has been shown to aid keratinocyte migration [30]. Silencing of EPPK1 upsets the intermediate filament network in keratinocytes, due to its role in lateral bundling of keratins [2]. In EPPK1-null mice, the corneal epithelium showed fragility against mechanical intervention and increased migration-dependent wound healing, with decrease in cell proliferation and E-cadherin expression [30]. In the liver, after stress or injury, EPPK1 is suggested to have a chaperon role to re-organise keratin networks. Similarly, EPPK1 may potentially protect against, and respond to, stress in the pancreas [31].

### Plectin (PLEC)

PLEC, a 500 kDa protein is expressed in a large variety of cell types [2]. The protein constitutes of a number of domains organized in three major segments [11]. The N-terminal region consists of an actin-binding domain (ABD), formed by two calponin homology domains, trailed by a plakin domain [11]. The actin binding domain binds to integrin α6β4, nesprin, F-actin and dystrophin [5, 32]. The central rod domain homodimerizes to form coiled-coil interactions. However, most of the rod domain is absent in a natural rod-less splice variant which retains the PLEC protein functions [3]. The C-terminal domain consists of six plakin repeat domains which includes nine spectrin repeats (SR1-SR9) and a SH3 domain [32]. The C-terminal domain of plakin mediates binding to intermediate filaments such as vimentin [32], while the plakin domain harbours interaction with integrin α6β4 [33] and BPAG2 (or type XV11 collagen) [11]. In muscle cells, PLEC can bind β-dystroglycan [34] and intermediate filament β-synemin [35], and the kinase Fer [36] in fibroblasts. The binding sites of these proteins are yet to be identified.

PLEC is a major intermediate filament cytolinker, which stabilises the cell cytoskeleton through keratin rearrangement, maintains actin filament dynamics and serves as a scaffolding base for signalling molecules [37]. It also links the nuclear envelope and centrosomes, while its long rod enable cells to maintain specific localisation of interacting molecules [32]. The actin and tubulin (microtubule) networks interact and crosstalk with the intermediate filament network via the actin binding domain (ABD) of PLEC1c [2, 37]. The phosphorylation sites of PLEC occur mainly at its C-terminus end [37]. Across the plakin family, the phosphorylation pathways are still being investigated, but so far, associations with differentiation, cell mitosis and migration have been revealed [2].

PLEC has many isoforms, arising from more than twelve first exon alternatives, giving N-termini variations, affecting binding sites and cellular location [11, 37]. In cells of mesenchymal origin, plectin1 (PLEC1) is a significant isoform included in connective and vascular tissues, eye lens and white blood cells [37]. In muscle cells, PLEC1 is responsible for linking the nuclear and endoplasmic reticulum membrane to the intermediate filament network. This may be through nesprin-3, an outer nuclear membrane protein and its binding partner torsin A linking to PLEC1 [37]. In comparison, in connective tissues and others, isoform PLEC1b specifically targets mitochondria and potentially forms a signalling platform and manages the organelle shape through its linking to the intermediate filament network [38].

PLEC appears to be associated with several cell-signalling axes, particularly its association with the keratin organisation of cytoskeleton modulates mitogen-activated protein kinase/extracellular signal regulated kinases (MARK/Erk) pathway. PLEC deficient keratinocytes, with no links between keratins and integrin α6β4, have increased potential for migration [39]. Deletion of PLEC enables its disassociation with integrin α6β4, which trigger Erk activation with a resultant migratory behaviour [39, 40].

PLEC has been shown to interact with RACK1 (receptor for activated kinase C), thus modulating the protein kinase C (PKC) signals and influencing the MAPK/Erk pathway [40]. In the absence of PLEC, RACK1 has been shown to move to the plasma membrane, from the peri-nucleus, where it affects PKC and also c-Src signalling, similar to that of fibroblasts and keratinocytes stimulated via external signals such as epidermal growth factor (EGF) [37] resulting in increased migration potential [39].

PLEC has also been associated with binding and modulating the proto-oncogene tyrosine-protein kinase FER and the energy-controlling AMP-activated protein kinase [36, 40]. It also has influence on the Rho/Rac/cdc42 family of small GTPases through its binding and rearrangement of actin filaments [41]. If apoptosis is induced by CD95 or tumour necrosis factor (TNF), PLEC is an early substrate of caspase 8 [42]. PLEC through its interaction with different signalling partners affects cellular behaviour such as proliferation, migration and invasion [43–45].

In a murine model, PLEC1 (mesenchymal isotype only) deficient T cells isolated from lymph nodes of PLEC1 null mice have reduced chemotactic migration in vitro and reduced leucocyte infiltration during wound healing in vivo [46]. These PLEC1 null mice have a normal lifespan, compared with the total PLEC null mice (no isotypes) which do not survive after birth due to skin blistering [46].

### Spectraplakins

The spectraplakins are a complex family with important cytolinker roles similar to both plakin and spectrin families. It is agreed that the spectraplakins distinctly include MACF1a and 1b, microtubule-actin cross linking factor 1, also known as actin cross-linking factor 7 (ACF7), and BPAG1a and 1b, bullous pemphigoid antigen 1 (from the disease where it was first identified), also known as BP230, often called dystonin (DST). They share similar features to BPAG1e (epithelial), BPAG1n (a neuronal isoform) and PLEC. The major function of the spectraplakins is their ability to bind any of actin filaments, intermediate filaments and microtubules [3, 47, 48].

Diversity in spectraplakin structure is generated by three to four alternative first coding exons. Their N-terminal actin-binding domain, comprised of two calponin homology domains, is also present in PLEC and neuronal BPAG1n. This is subsequently followed by a protein binding plakin domain present in all plakins and spectraplakins, except EPPK1. MACF1b and BPAG1b have a centrally located intermediate filament binding in the PLEC repeat domain (sometimes referred to as plakin repeat domain, PRD). PLEC, BPAG1e and BPAG1n have this PLEC repeat domain at their C–terminus, as does DSP and EVPL. All spectraplakins have a large quantity of alpha-helical ‘spectrin repeats’ which form a large rod and contribute to their bulk. They also all have a calcium binding site, two alpha helices linked by a short loop region, called an EF hand domain, due to its similarity to the third (E to F) calcium binding site in parvalbumin [49]. This is followed by a growth-arrest-specific 2-related (GAS2) domain and C-terminus that makes up their microtubule binding capacity [2, 3, 48].

Despite their multiple binding sites, spectraplakins appear to crosslink just one element of the cytoskeleton most of the time, but when necessary, such as in axon growth, binding of actin and microtubules occurs simultaneously. Loss of spectraplakins creates chaos in the cytoskeletal networks, which affect many cellular functions such as adhesion, polarisation, stabilisation and positioning of the nucleus and organelles and even interrupting intracellular transport [3, 48].

## Plakins in non-cancerous disease

The plakins are linked to several diseases, mostly involving the skin, muscle and the neurons. Many of the plakins were first described from investigation of the autoimmune conditions, paraneoplastic pemphigus (blistering, inflamed skin and mucosa with co-presenting neoplasm) and bullous pemphigoid (large, sub-epidermal blistering). Changes in protein expression or function through genetic changes often leads to the skin condition epidermolysis bullosa simplex (EBS), a skin blistering condition with hyperkeratosis of hands and feet. MACF1 is not associated with diseases in humans, but studies in mice have found an absence of MACF1 results in embryo death during gastrulation [47]. Generally, genetic deletions of plakin proteins results in abnormal immune responses leading to diseases that results from the development of autoantibodies against plakins [2, 3]. Table 1 demonstrates a list of the currently known genetic and autoimmune pathologies resulting from loss or damage of the plakin proteins.

**Table 1:** Tissue distribution of plakins in normal, genetically compromised and autoimmune diseases

Name/Alias	Size	Tissue distribution	Normal function, localisation and binding partners	Disease
Plectin (PLEC)	> 500 kDa	Wide ranging, including skin, muscle, nervous system, GI tract	Hemidesmosomes, Outer nuclear/endoplasmic reticulum, (PLEC1) muscle Z disks (PLEC1d) mitochondria (PLEC1b) sarcolemmal dystrophin-glycoprotein complex (PLEC1f) Intermediate filaments, microfilaments, microtubules-different isoforms have tissue specific functions	Genetic (abnormal protein)—epidermolysis bullosa simplex (EBS), pyloric atresia, myopathy (ophthalmoplegia, cerebral atrophy) Autoimmune (auto–antibodies)—paraneoplastic pemphigus, bullous pemphigoid
Desmoplakin (DSP)	DSPI 322 kDa DSPII 259 kDa	Heart, stratified epithelia, DSPII found in tissues other than heart and simple epithelia, otherwise widely expressed	Desmosomes (most abundant desmosomal protein) Intermediate filaments, intercalated disks	Genetic (abnormal protein)—palmoplantar keratoderma, woolly hair, cardiomyopathy, lethal acantholytic epidermolysis bullosa Autoimmune (auto–antibodies)—paraneoplastic pemphigus
Envoplakin (EVPL)	210 kDa	Skin and other stratified epithelia	Cornified envelope Intermediate filaments	Genetic—not known Autoimmune (auto–antibodies)—paraneoplastic pemphigus
Epiplakin (EPPK1)	450–700 kDa	Skin and other stratified epithelia	Cornified envelope Intermediate filaments	Genetic—not known Autoimmune (auto–antibodies)—blistering diseases
Periplakin (PPL)	195 kDa	Skin and other stratified epithelia	Cornified envelope Intermediate filaments	Genetic—not known Autoimmune (auto–antibodies)—paraneoplastic pemphigus
Microtubule-actin cross linking factor (MACF1),	~ 600 kDa	Wide ranging	Microtubules, microfilaments	unknown
Bullous pemphigus antigen1 (BPAG1)	1a ~ 625 kDa 1b ~ 834 kDa 1e 230 kDa	Wide ranging but 1a/1n nervous system 1b muscle 1e stratified epithelia	Microtubules, microfilaments Hemidesmosomes, intermediate filaments	1a/1b—genetic (abnormal protein)—lethal form of dysautonomia psychomotor retardation, autoimmune—not known 1e—genetic (abnormal protein)—EBS, autoimmune (auto–antibodies)—bullous pemphigoid, paraneoplastic pemphigus

## Plakins in cancer

### Role of DSP in cancer

DSP is a desmosomal protein involved in cell–cell adhesion [50]. Reduced expression of DSP has been noted to increase invasion and metastasis in several cancers [51, 52]. These changes in DSP expression occurs following epithelial-mesenchymal transition (EMT), an essential biological process observed during embryogenesis and wound healing [53]. However, EMT in cancer involves downregulation of the expression of desmosomal, adherens/tight junction and cytolinker proteins such as E-cadherin, occludens, claudins, EpCAM, α6β4 integrin, different cytokeratins, DSP, PPL. The process also involves simultaneous upregulation of the expression of intermediate filament and extracellular matrix (ECM) associated proteins such as vimentin, fibronectin, N-cadherin, β1, β3 and β5 integrins and matrix metalloproteases (MMPs). These changes in desmosomal, adherens/tight junction, cytolinker and ECM proteins is necessary for the transformed cells to undergo epithelial-mesenchymal morphological changes to facilitate motility for dissemination [54].

Reduced expression of DSP, plakoglobin and plakophilin desmosomal proteins was noted in triple negative breast and other epithelial cancers [16, 55, 56]. This occurred concurrently with enhanced expression of EMT-inducing transcription factors such as Slug, Sip1/ZEB2 (zinc finger E-box-binding homeobox 2)[16, 55, 56]. In addition, reduced expression of DSP and tight junction proteins was also noted in pancreatic tumour cell lines, which had undergone EMT [52]. Down-regulation of DSP and E-cadherin was induced in prostate cancer cell line PC3, in response to upregulation in microRNA (331-3p) that concurrently upregulated EMT markers, such as N-cadherin, vimentin and Snail, suggesting that DSP loss occurs with the initiation of an EMT process in transformed cells [57]. Similarly, increased expression of EMT markers, N-cadherin and fibronectin in response to greater activity of EMT promoters Slug and Snail, concurrent with reduced levels of DSP and occludin was observed in highly migratory human pancreatic cancer cell lines compared to less aggressive cell lines [58].

Further to the above studies, alteration in the expression of DSP has been observed with the differentiation of oral pharyngeal carcinomas, where the expression of DSP in differentiated tumours that produced distant metastatic tumours within 3 years of follow up was markedly higher than in undifferentiated tumours [59]. Consistent with that, loss of DSP expression in head and neck squamous cell carcinoma (HNSCC) correlated with the loss of differentiation of primary tumour cells and presence of lymph node metastases [59, 60]. In addition, investigation of pre-cancerous dysplastic oral epithelium detected changes in DSP immunoreactivity, suggesting that desmosomal adhesion disruption is an early event in the progression of oral squamous cell carcinoma [59, 60]. Similarly, exploration of the progression of squamous intra-epithelial lesions to squamous cell carcinoma of the cervix, demonstrated increasing levels of DSP inhibition with increasing severity of disease [61, 62]. Using genetically modified mice in pancreatic neuroendocrine tumours changes in DSP and other desmosomal protein expression is an early event in the tumourigenic process and preludes changes in the expression of adheren junction proteins and tumour cell invasion [52].

DSP expression levels have shown to vary across a range of lung cancers [51, 63, 64]. In adenocarcinoma and adenosquamous carcinoma of lung minimal expression of DSP was noted [63]. Consistent with that, using an induced overexpressing DSP model of non-small cell lung cancer (NSCLC), the tumour-suppressive function of DSP behaviour was demonstrated through the inhibition of the Wnt/β-catenin/TCF/LEF (transcription factor) pathway [51]. In that context, epigenetic silencing of DSP was observed in primary lung tumours and cell lines [51]. Contrary to that, increased DSP expression was observed in lung squamous cell carcinoma, a subset of NSCLC, which displayed increased expression and distinct distribution of other desmosomal proteins including integrin β4. DSP expression was highest in the centre of the tumours, where the most differentiated cells were found [64].

Estrogen (E2) is suggested to have a role in modulating desmosomal protein expression and desmosome formation. Exposure of normal and malignant mammary cells to estrogen for prolonged periods resulted in an increased expression of desmosomal proteins including DSP [65]. However, a reduction in DSP expression was observed after partial inhibition of the estrogen receptor α [65]. Silencing of DSP expression by siRNA resulted in the prevention of E2-dependent cell adhesion, indicating there is a functional relationship between estrogen receptors, DSP and desmosome formation and thus cell adhesion. The modulation of desmosomes by estrogen and its receptor could help maintain epithelial tissue integrity and explain the lower invasiveness seen in ERα positive breast tumours [65].

The above studies indicate that DSP may have a tumour suppressive role in cancers and its expression may be reduced by the induction of EMT at an early stage of cancer progression. However, the tumour suppressive role of DSP in cell context dependent and relies on the differentiation status of the tumour, as enhanced expression of DSP is observed in differentiated tumours [63, 64, 66].

### Role of EVPL, PPL in cancer

EVPL, PPL and involucrin null mice showed skin fragility but also a significant resistance to developing skin tumours when challenged with tumour-stimulating TPA (12-o-tetradecanoylphorbol-13-acetate) [67]. These mice responded with increased levels of chemokines and cytokines (type 2, type 17) resulting in the recruitment of mast cells, granulocytes and CD4 + T cells which may lead to immune editing of tumour cells. The tumour protective mechanisms in these null mice also involved signalling between Rae-1 expressing keratinocytes and the natural killer cells having 2D receptor (NKG2D) [68]. These results suggests that absence of ENVPL, PPL and involution may activate the immune system through regulation of certain cytokines/chemokines.

In cancer, reduced expression of PPL and EVPL have been observed in oesophageal cancers [69], and the cellular localisation of PPL was noted to change with disease progression. Using immunohistochemistry, PPL expression was observed at cell–cell boundaries of normal oesophageal epithelium and dysplastic lesions, whereas it relocated to the cytoplasm in early cancers and was scarcely expressed in advanced tumours [69]. In patients with a history of smoking and who present with hyper-methylation of oesophageal mucosa, DNA methylation of PPL promoter sequences leads to reduced expression of PPL and have been linked to the development of oesophageal squamous cell carcinoma [70], suggesting that loss of PPL may be one of the early events in the progression of oesophageal cancer.

Consistent with oesophageal cancer, in urothelial carcinoma of the urinary bladder, the expression of PPL was reduced with increasing pathological stages [71, 72]. Serum PPL studies of urothelial carcinoma patients indicated higher levels of PPL in muscle-invasive carcinoma, than non-muscle invasive carcinoma. However, both types of carcinoma had lower serum PPL levels than healthy controls, contributing to the pre-operative evaluation of these conditions [72]. These observations are consistent with the downregulation of PPL expression in colon carcinomas (which undergo EMT) compared to normal and para-carcinoma tissues [73].

PPL also plays a role in the metastasis of triple-negative breast cancer (TNBC) into the brain [74]. Reduced PPL expression has been observed in brain metastatic lesions of TNBC patients. In TNBC cell lines, reduced expression of PPL by siRNA, resulted in reduced cell migration and invasion, but had increased cell growth in soft agar, suggesting that reduced expression of PPL in brain metastasis may be important for the growth of tumours in TNBC patients [74].

In vitro experiments on cancer cell lines have shown that, the expression of PPL have varied results on cellular functions. The absence of PPL expression hinders collective migration and wound closures in epithelial cancer cells grown as monolayers as PPL facilitates the reorganization of keratin filaments at the edge of wounds [75]. Consistent with that, downregulation of PPL expression in vitro resulted in reduced cellular proliferation, adhesion and movement, linked to G0/G1 cell cycle arrest and loss of activation of PAktSer473 kinase via the PI3 kinase pathway in pharyngeal squamous cell carcinomas [76]. On the contrary, in a colon cancer cell line model (HT29), increased proliferation, migration, invasion and EMT initiating ability was noted in response to PPL knockdown [73]. However, the process was reversed in terms of decreased proliferation, migration and EMT ability when PPL was overexpressed in the same cell line, strongly suggesting an inverse relationship of PPL expression and induction of EMT in colon cancer cells [73]. Decreased proliferation in response to PPL overexpression was due to higher rate of G1/G0 cell cycle arrest resulting from increased expression of CDK inhibitors p21, p27kip and p-Rb [73]. PPL knockdown in that model partly reversed the G0/G1 cell cycle arrest induced by PPL overexpression. These observations suggest that the effect of PPL expression on the function of cancer cells varies and is tumour context dependent, regulated by autocrine or paracrine factors that modulates cell cycle properties.

### Role of EPPK1 in cancer

Both PLEC and EPPK1 are expressed in duct cells and centroacinar cells of the mature pancreas, with increasing expression of both in pre-cursor lesions and pancreatic ductal adenocarcinoma [2]. EPPK1 expression is increased in early pancreatic intraepithelial neoplasia (PanIN) but decreased levels were noted in more developed disease [77]. EPPK1 has a potential role in the EGF (epidermal growth factor) signalling pathway as its binds to the EGF receptor [78]. The participation of EPPK1 in EGF signalling was reported in pancreatic development and carcinogenesis [77, 79]. Activation of fluorescent-labelled EPPK1 in HeLa cells, originally of cervical cancer origin [29], demonstrated dynamic movement of EPPK1 protein, from one side of the cell membrane to another during migration. In HeLa cells increased migration was observed when EPPK1 expression was suppressed by knockdown while decreased migration was noted in EPPK1 overexpressed cells [29]. In 3D cell spheroids, EPPK1 was expressed in the outermost cell layer and barely detected in the interior of the spheroids, suggesting a role of EPPK1 in epithelial cell polarisation and spatial organisation [29]. However, in 2D cultures EPPK1 stabilised the keratin networks via colocalization with zonula occludens-1 (ZO-1), a marker of tight junctions, and inhibited the motility of cells by reorganizing the actin filaments [29].

### Role of PLEC in cancer

Plectin expression was significantly higher in the SW480 colon cancer cell line than the lower grade HT29 colon cancer cell line [80]. In SW480 cells, in vitro suppression of PLEC by siRNA inhibited actin dynamics at scratch wound edges and reduced invasion, migration and adhesion of these cells [80]. Re-introduction of only the actin-binding domain of PLEC was sufficient to re-instigate the actin assembly at the scratch wound edge [80]. Studies have also shown that increased PLEC and vimentin expression, through PLEC complex regulation of vimentin assembly, correlate with invasive phenotypes in bladder cancer and invasion and metastasis in androgen-independent prostate cancer [43, 81]. PLEC1 expression is upregulated in oesophageal squamous cell carcinoma (SCC) and is a likely biomarker in this disease [82]. High PLEC expression has been noted in head and neck squamous cell carcinomas (HNSCC) and has been associated with increased recurrence and decreased survival rates in patients [45]. Decreased expression of PLEC by siRNA suppressed the invasion, migration and proliferation of HNSCC cells and downregulation of the Erk1/2 pathway [45]. It has been postulated that PLEC may contribute to cell migration, proliferation and invasion through its association with integrin β4 subunit, resulting in the Erk1/2 activation [2, 45].

PLEC biology has been studied significantly in pancreatic cancers [83, 84]. PLEC1 has been identified as a biomarker of sufficient sensitivity and specificity for cystic fluid analysis in the early diagnosis of intra-ductal papillary mucinous neoplasms (IPMN), a group of lesions with different metastatic potential, detected by computed tomography (CT) scan [83]. PLEC1 expression enhances during the development of pancreatic intraepithelial neoplasia, PanIN stage II to PanIN stage III, precursor lesions of invasive and metastatic pancreatic ductal adenocarcinoma (PDAC) [83–85]. Later in disease progression, PLEC expression changes from its cell membrane localisation to a diffuse cytosolic distribution [84].

PDAC cells produce exosomes, which produce pre-metastatic niche environments in other tissues, such as liver [85]. These exosomes are enriched with PLEC and integrin β4, which is necessary for PLEC inclusion [85]. Incubating PLEC-rich exosomes with cell lines devoid of cell-surface PLEC can induce abnormal cell-surface expression of PLEC [86]. Additionally, in non-PDAC cell lines, induced over expression of PLEC1a and 1f resulted in these isoforms being located on the cell surface [86]. Normal keratinocytes, even though expressing PLEC1a, 1f and integrin β4, do not produce exosomes [86]. In PDAC, suppression of PLEC results in reduced proliferation, invasion and migration, and inhibits exosome formation. It is however, unclear whether exosome formation is stimulated by intracellular or exosomal PLEC [86].

PLEC is down regulated in hepatocellular carcinoma [44, 87]. Further to that, in hepatocellular carcinoma (HCC) cell lines, PLEC deficiency results in irregular loosened bundles of intermediate filaments leading to observable pleomorphism [87, 88]. Induced PLEC deficiency in healthy hepatocytes also showed augmented cytoskeletons, through the altered expression and rearrangement of cytokeratin 18 (CK18). PLEC’s role in the spatial organisation and anchorage of the cytoskeleton is phosphorylation-dependent, as PLEC’s coordination of lamin B and vimentin is modulated by protein kinase A and protein kinase C [35]. In addition, the breast cancer susceptibility protein, BRCA2, interacts with PLEC [89], where the BRCA2/PLEC complex is involved in nuclear duplication and centrosome formation. During the M cycle, cyclin dependent kinase 1/cyclin B kinase (CDK1/CycB) actively phosphorylates PLEC, interrupting its binding of intermediate filaments and initiating network disassembly [90]. This is followed by centrosome movement resulting in perinuclear localisation potentially due to PLEC/BRCA2 complex interaction with the centrosome [89, 90].

## Ovarian cancer

Ovarian cancer is an aggressive and progressive gynaecological neoplasm and carries a poor prognosis [91]. It is the fifth most prominent cause of cancer-related deaths amongst women worldwide [92]. At diagnosis, in majority of the cases, the cancer is manifested by an extensive intra-abdominal spread that involves peritoneum and the surrounding organs [93]. Even though extra-abdominal metastasis at diagnosis is rare, in sporadic cases that may involve metastasis to thyroid, bone, heart, breast, colon and brain [93–95]. However, mortality from ovarian cancer occurs mainly from intra-abdominal spread and death from distant metastasis is uncommon [93–95].

Despite recent advances in conventional and targeted chemotherapy, precision with debulking surgery, and the search for the elusive and reliable early diagnostic test, the five-year mortality rate of ovarian cancer patients still remains as high as 60–70% [96]. To make this challenge more complex, recent findings have classified ovarian cancer not as a single disease but as a mix of genetically different ovarian neoplasms [97]. Histologically, three main types of ovarian neoplasms have been shown to persist; cancer arising from epithelial cells or germ cells or sex cord stromal cells (hormone secreting, supporting, stromal cells within ovary) [98]. The most aggressive and common (~ 90%) of these neoplasms is the epithelial ovarian cancer which is further divided into four histological sub-types commonly known as mucinous, endometrioid, clear cell and serous carcinomas [97]. Among these cancers, the serous subtype constitutes nearly 80% of epithelial ovarian cancers [97].

Traditionally the ovarian surface epithelium (OSE), a single layer of epithelial cells lining the ovary, was considered as the cell of origin for serous ovarian tumours [99]. These tumours were shown to arise from OSE, which are damaged by the inflammatory cytokines and reactive oxygen species generated during the ovulation process [100]. Most of the damaged OSE is repaired during the ovulation cycle before the commencement of the next cycle [101]. However, some damaged cells persist and accumulation of these over time may lead to malignant transformation in these cells [102, 103]. It has been postulated that under the ovulation-induced inflammatory conditions, the damaged unrepaired OSE cells may become entrapped in cyst-like structures commonly known as ‘cortical inclusion cysts’ (CIC) which are thought to be the origin of ovarian cancer [101]. This OSE-CIC theory relating to the origin of epithelial ovarian cancer is consistent with epidemiological data, which associates low risks of ovarian cancer in women who undergo less number of ovulatory cycles (due to pregnancy, lactation or intake of contraceptive pills) [104]. However, the theory lacks explanation for the presence of genetically diverse peritoneal carcinomas, which does not justify a CIC origin [97].

In the last fifteen years, extensive immunohistochemical analysis of the Fallopian tubes obtained during salpingo-oophorectomy from women with inherited mutation in germline breast cancer susceptibility proteins type 1,2 (BRCA 1,2) have shown that the fimbriae end of the Fallopian tube that expresses serous tubal intraepithelial carcinoma (STIC) lesions, to be a potential source of high-grade serous ovarian tumours [105, 106]. Women with BRCA1,2 mutations are predisposed to breast/ovarian cancer syndrome and carry a lifetime risk of 60–80% for breast cancer and 40–50% ovarian cancer respectively [107]. They develop dedifferentiated, aggressive and invasive triple negative breast cancer and high-grade serous ovarian cancer, which carry a poor prognosis [108]. The cells within STICs have a high proliferative index (indicated by high Ki67 expression) and a ‘p53 signature’ (mutated, non-functional p53) and exhibit the DNA damage marker γ-H2AX [109] indicating damaged DNA double strand breaks [110]. Later clinical studies have shown that 38% of this signature persists in women with BRCA1,2 mutations and 80% of that occurs in STICs located at the fimbriae end of the Fallopian tube, potentially identifying the Fallopian tube as the origin of high-grade serous ovarian cancers [111, 112].

### Initiation and progression of ovarian cancer

The pathophysiological mechanisms of intra-abdominal spread in ovarian cancer involves a few critical steps and has been studied extensively in the last few years [113]. Metastasizing tumour cells from solid tumours may directly invade the adjacent intra-abdominal organs or may disaggregate from solid tumours and accumulate in ascites (tumour fluid) [114]. In cases of direct intra-abdominal spread these disaggregated cells floating in nutrient enriched ascites survive, either as single cells, or mostly by clustering as multicellular aggregates of cells commonly called ‘spheroids’ [114, 115]. Malignant ascites with free-floating single tumour cells, spheroids, immune, endothelial and stromal cells are often observed in ovarian cancer patients at diagnosis and are a common scenario in most recurring patients [96]. Increased vascular permeability and blockage of peritoneal lymphatic drainage by the disseminated cancer cells in the peritoneum is a common known cause of ascites formation [96]. Recent research indicates that the interaction between disseminated tumour cells and the peritoneal mesothelial cells play a critical role in ovarian cancer dissemination [116]. In that scenario, the shear and compressional pressure induced by ascites alters the mesothelial cell lining of the peritoneum by initiating the formation of ‘tunnelling nanotubes’ (TNT) on the surface of peritoneal mesothelial cells, which facilitate the transfer of mitochondria from mesothelial cells to ovarian cancer cells [117]. The growth factors and other soluble molecules in ascites also induce EMT in mesothelial cells by a process commonly known as mesothelial mesenchymal transition (MMT) which may retract the peritoneal mesothelial lining to promote the implantation of tumour cells into the peritoneal stroma [116]. However, a recent study has shown that the mesothelial cells lining the peritoneum remain intact but undergo senescence due to loss of adherent junction proteins (connexion 43, E-cadherin, occludens, desmoglein) that fosters invasion of cancer cells through the mesothelial lining to the sub-mesothelial matrix of the peritoneum to form secondary lesions [116, 118]. This cross talk between the invading cancer cells and the peritoneal mesothelial cells is facilitated by TNTs that support the transport of mitochondria from peritoneal cells to cancer cells to promote cancer cell growth [116]. Previous studies have shown that environmental stress facilitates the formation of TNT in different types of cancer cells to reprogram metabolism in stressed cells for increased production of ATP to stimulate survival [119, 120]. In addition, TNT-induced transfer of mitochondria from bone-marrow stromal cells to myeloid leukemic cells or endothelial cells to cancer cells during chemotherapy treatment has been shown to promote survival of resistant cancer cells [121, 122]. These observations suggests a unique role of ascites-induced compressive pressure in promoting intra-abdominal metastasis in ovarian cancer.

### Epithelial mesenchymal transition (EMT) and mesenchymal to epithelial transition (MET) in ovarian cancer progression

It has been postulated that EMT may initiate the early precursor lesions for high-grade serous ovarian cancer. The presence of TGFβ and inflammatory cytokines/growth factors in the follicular fluid released during the ovulation may initiate EMT in the secretory cells of Fallopian tubes or the cells lining the CICs of OSE [102]. An association between BRCA1 and EMT has been established in breast cancer [123]. A loss of BRCA1 in mammary epithelial cells results in dedifferentiation of these cells with upregulation of CD44high/CD24low cancer stem cell (CSC) phenotype and induction of EMT [124] However, the status of BRCA mutation and EMT remains unexplored in ovarian cancer.

The EMT process has been associated with peritoneal metastasis, progression-free and overall survival in ovarian cancer, suggesting that EMT is intricately involved with ovarian cancer dissemination and therapy resistance [125]. A recent study of 174 primary ovarian and 34 metastatic tumours suggested EMT was a poor prognostic indicator for ovarian cancer by associating low E-cadherin and high Snail expression with high peritoneal dissemination, low overall and progression-free survival in patients [126]. Ascites-derived tumour cells obtained from ovarian cancer patients were shown to undergo EMT during aggregation into spheroids, and this phenomenon was reversed when these cells were allowed to adhere on substratum in monolayer cultures [127]. E-cadherin expression was significantly reduced in aggregating spheroids compared to adherent cells in association with significant upregulation of transcription factors such as Snail, Twist and Zeb2 [127]. Studies have also suggested that spheroids have heterogeneous cadherin expression and enhanced E-cadherin expression is observed in established, cohesive and spherical spheroids which are difficult to disaggregate [128, 129]. These spheroids tend to preserve their free-floating abilities in the ascites microenvironment, and metastatic dissemination only occurs if a gain in N-cadherin expression is attained through EMT process. The process of EMT is induced by the hypoxic ascites microenvironment which activate hypoxia-induced factor 1-alpha (HIF1α) with consequent increase in the transcription of Snail resulting in EMT, increased cell motility and invasion on the sub-mesothelial layer of the peritoneum [130, 131]. However, once the cells have migrated and colonization has occurred the process of EMT is reversed through MET for the secondary lesions to establish at a distant site [103, 132]. This continued transitional dynamics of ovarian cells between EMT and MET contributes to the metastasis and invasion of cancerous cells from primary tumours to secondary sites, and in rare cases dissemination via blood, lymph, and then invasion into other tissues [133, 134].

Recent studies indicate that ovarian cancer cells can exist in an intermediate ‘partial or hybrid EMT (E/M)’ state with characteristics of both epithelial and mesenchymal cells [132, 135]. Cells in E/M state possess a superior advantage for survival and metastasis compared to cells in either epithelial or mesenchymal state as they can readily differentiate towards either epithelial or mesenchymal state depending on the stimulus received from the tumour microenvironment [136]. Hence, E/M cells are more adaptable to migration, colonization at distant sites and are enriched in therapy-resistant CSCs, which retains the capacity for self-renewal as well as production of differentiated progenies to generate the bulk of tumours at metastatic sites or as recurrent/relapsed tumours after therapeutic treatments [137]. It is postulated that the alterations in E/M or EMT/MET phenomenon in intraperitoneal ovarian tumour cells is not due to any genetic mutation but is likely due to the origin of ovarian cancer itself which arises either from ovarian surface epithelium or differentiated columnar epithelial cells both of which contain epithelial and mesenchymal traits. It may also result from the external stimuli received by the cancer cells in the ascites microenvironment [138]. Ascites contain substantial amounts of growth factors such as lysophosphatic acid (LPA), transforming growth factor-β (TGFβ), epidermal growth factor (EGF), hepatocyte growth factor (HGF), interleukins (IL)1β, IL-6, IL-8, chemokine ligand 5 (CCL-5) and chemokine receptors (CCR)-1/3/5, CCL-19/21 and CCR-7 [139], with known EMT initiating roles in ovarian cancer [114, 140].

In a recent study, immunostaining of ovarian cancer cell lines showed heterogeneous mixture of cells containing hybrid Ecad + /Ncad + clones or homogenous only Ecad + or Ncad + clones [128]. However, the hybrid Ecad + /Ncad + clones showed greater proliferation than homogenous Ecad + or Ncad + clones, indicating once again the greater role of a E/M phenotype in facilitating ovarian cancer growth [128]. In addition, E/M cells involved with collective migration are enriched in the outer layer of multicellular spheroids [138], coincided with a recent study which showed KRT14 + leader cells enriching the outer edge of multicellular spheroids and collectively producing actin-rich invapodia to displace mesothelial cells for peritoneal invasion [141]. Loss of KRT14^+^ cells diminished the invasive capacity of ovarian cancer spheroids, suggesting a potential role of E/M KRT14 + cells in peritoneal invasion [142].

### Drug resistance, recurrence and CSCs in ovarian cancer

Current treatment for ovarian cancer consist of surgery to remove the tumour (debulking) and any associated ascites, followed by chemotherapy. This usually results in significant reduction of tumour burden, but subsequent recurrence of tumour growth is common. Responsiveness of tumours to chemotherapy can be linked to tumour grade [143]. Low-grade tumours tend to be slower growing and less responsive to current chemotherapy, but potentially react to hormone-based treatments [143]. The high-grade tumours are more responsive to chemotherapy initially, but not hormones, with increasing lack of response to chemotherapy leading to chemo-resistant recurrent disease [143]. Current chemotherapies for ovarian cancer patients include a combination of platinum based (DNA cross-linking) and taxane based (microtubule/mitosis interference) drugs, to induce apoptosis in the bulk of cancer cells within the tumour [144]. However, a small population of resistant cells with properties of CSCs evade cancer treatments and they reinitiate cancer regrowth [145–147]. Chemotherapy has limited effectiveness against CSCs, due to the slow replication potential of these cells, high expression of efflux channels such as the ATP-binding cassette (ABC) transporters, high response to DNA damage and repair and their ability to avoid host immune system [148, 149]. These essential plastic properties of CSCs are crucial for tumour relapse and progression and are critical for the development of CSC-specific strategies in combination with standard chemotherapies [144, 149, 150].

In ovarian cancer identification of CSCs have proved challenging as CSC markers identified in other tumours such as CD44, CD117, EpCAM, ALDH1, CD28 and OCT4 [148, 149, 151, 152] are not consistently identifiable in all ovarian tumours. These markers are not uniformly displayed [153], and this plasticity is influenced by the evolving tumour microenvironment [154]. In this context, it has been shown that each population of CSCs carries inherent functions within the tumour and different pools of CSCs have varied functions, which are not consistently expressed within the tumours [154]. Adding to this complexity, patients have multiple pools of CSCs within each tumour expressing different markers making specific targeting of CSCs difficult [154]. In addition, the process of EMT collaborates with CSCs, and treatment with platinum-based chemotherapy can induce EMT and CSCs in chemotherapy-treated residual cancer cells [150, 155]. Several signalling pathways can facilitate the initiation of CSCs in ovarian cancer [154]. Among these PI3 kinase/Akt/PTEN [156, 157], Jak2/Stat3 [155], NFκB [158], Wnt [159], Notch [160] and Hedgehog [161] have been shown to facilitate tumour progression and chemotherapy resistance in ovarian cancer. Inhibiting these pathways in in vitro cultures has shown suppression of tumourigenesis and chemosensitivity in cell line and animal models [154, 155].

Breaks in double strand DNA occur in normal cells and in response to environmental factors such as ionising radiation [162]. Repairs to double strand breaks (DSB) are challenging for cells, with ineffective or incorrect repair leading to genomic instability [162, 163]. The BRCA1, 2 genes are involved in DSB repair, with inherited mutations in these genes causing specific defects in the DNA repair capacity of the cell [163]. Poly (ADP ribose) polymerase 1 (PARP1) is involved in the repair of single strand DNA repair. Inhibition of PARP1 can trigger ‘synthetic lethality’ in cells with BRCA1/2 mutations (and other faulty DSB repair mechanisms) [163]. The use of PARP inhibitors has been successful in ovarian cancers in both BRCA1/2 mutated and non-mutated patients [163–165]. This has resulted in increased progression-free survival in platinum resistant as well as sensitive patients who generally are liable to recurrence within the first few months of first line chemotherapy [163]. However, some patients undergo resistance to PARP inhibitors and ongoing genomic studies are in progress to understand that phenomenon [166]. A recent in vitro and in vivo study in ovarian cancer has demonstrated that resistance in PARP inhibitor is accrued through enrichment of CD117 and CD133 CSCs [167]. PARP inhibitor treated residual cells undergo G2-M phase cell cycle arrest, but enhance γH2AX, RAD15 and DMC1 foci leading to accelerated DNA repair mechanism [167]. Other studies in colorectal cancer [168] have shown that combination of PARP inhibitors with radiotherapy and chemotherapy sensitises CSCs to the effect of given therapy. Recent clinical trials in ovarian cancer, which includes PARP inhibitors in combination with Bevacizumab or chemotherapy, have shown promising results in terms of overall all survival and progression-free survival [164, 169–171].

### Plakins in epithelial ovarian cancer

Very little is known about the plakin biology in ovarian cancer. In terms of ovarian cancers, the influence of estrogen on DSP and desmosomes [65] correlates with metastasis and PPL expression [74]. As described above, even though the role of EMT has been studied extensively in ovarian cancer, the implication of plakin biology, intimately involved with EMT and metastasis remains unknown.

**Fig. 2:**
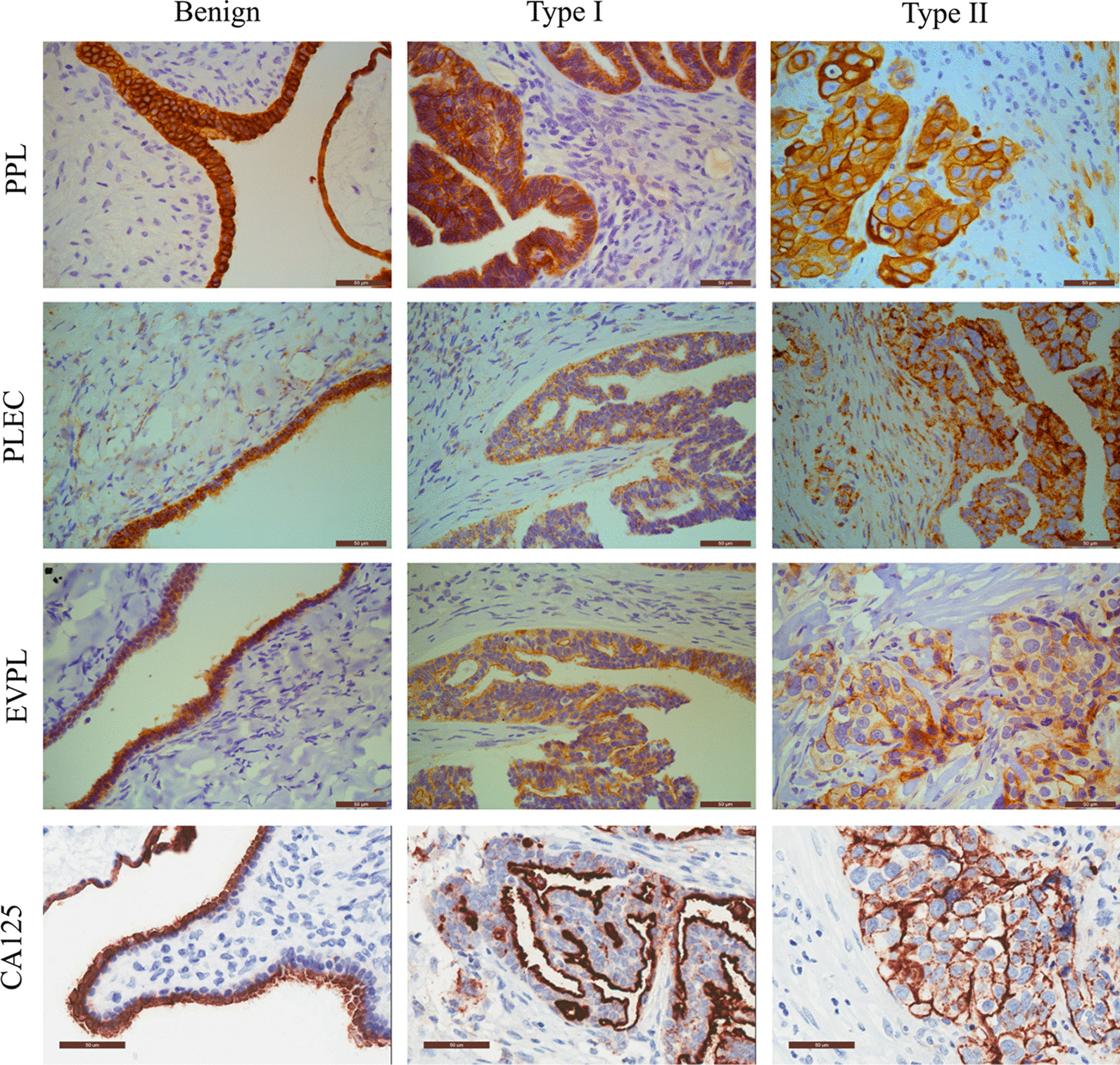
Representative immunohistochemical staining of PPL, EVPL, PLEC and CA125 on formalin fixed paraffin embedded (FFPE) serous ovarian benign, Type I (low grade) and Type II (high-grade) ovarian tumours. Immunohistochemistry images of FFPE sections representing staining of PPL, PLEC, EVPL and CA125 of benign, Type I (low-grade) and Type II (high-grade) ovarian tumours. Samples were obtained from patients diagnosed with ovarian cancer before surgery under protocols approved by the Human Research and Ethics Committee (Ethics approval #09/09) of the Royal Women’s Hospital, Melbourne, Australia after gaining patient's consent. Immunohistochemistry was performed as described previously [150, 155]. Sections were assessed microscopically for positive DAB (brown), haematoxylin (blue) counterstain staining. Magnification (40×), scale bar = 50um

In this review, we demonstrate that plakins (PLEC, PPL and EVPL) are expressed in benign, Type I (low-grade) and Type II (high-grade) ovarian tumours. In benign and Type I tumours, the expression of plakins are confined to the epithelial lining of the tumours (indicated by positive CA125 staining) (Fig. 2). However, in Type II tumours, the epithelial boundary is lost and the expression of plakins are distributed throughout the tumours, confined to the cluster of epithelial cells (indicated by positive CA125 staining) within the tumours (Fig. 2).

**Fig. 3:**
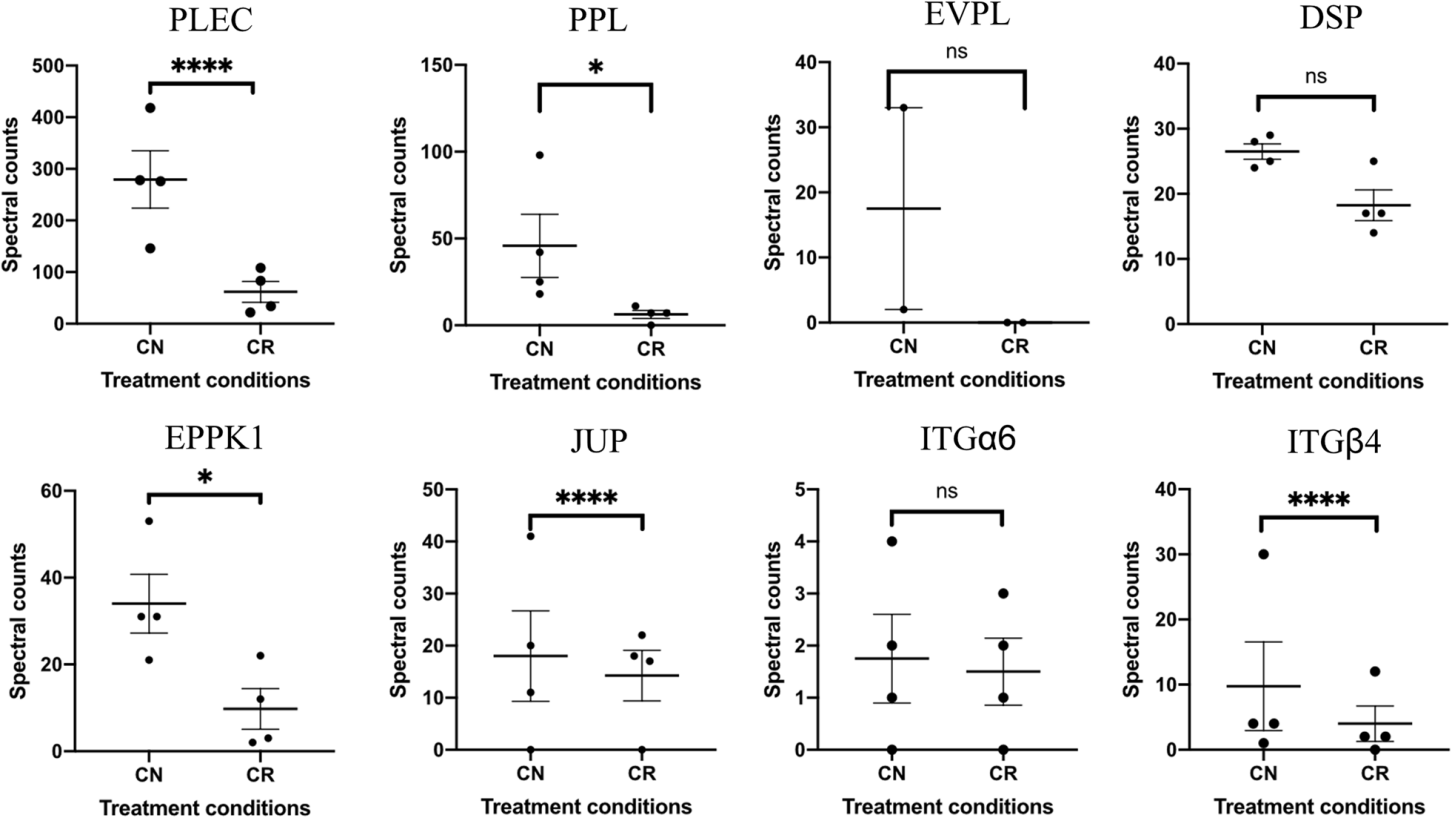
Proteomic based spectral counts of PLEC, PPL, EVPL, DSP, EPPK1, junction plakoglobin (JUP) and plectin-associated α6 and β4 integrin subunits in CN and CR ascites-derived ovarian cancer cells (± SEM, n = 4 for both CN and CR) previously described in proteomics study [172]. Statistical significance was determined by a Chi-square test and is indicated by *p < 0.05; ****p < 0.0001; ns, not significant

Our interest in the involvement of plakins in ovarian cancer was heightened by proteomics analysis of tumour cells derived from the ascites of chemonaïve (CN) and recurrent (CR) samples [172]. The CN samples were collected from ovarian cancer patients at diagnosis while the CR samples were collected where the disease progressed post chemotherapy treatment, between 6–20 months after first line of treatment. The tumour cells from these samples were isolated using a novel culturing technique (developed in our laboratory) without the contaminating stromal and immune cells [173]. The members of the plakin family that were differentially expressed between the CN and CR samples were PLEC, EVPL, PPL and EPPK1. Graphical representation of plakins and related desmosomal and hemidesmosomal associated proteins differentially expressed between CN and CR ascites-derived tumour cells [172] is provided in Fig. 3.

The study suggests that the expression of major plakins is lower in CR ascites-derived tumour cells compared to CN cells. The reason for the downregulation of plakins in CR versus CN samples is still unknown. However, it can be postulated that this may be due to long-term sustenance of the CR ascites-derived floating spheroids compared to CN spheroids. Long-term sustenance in the floating state may make proteins required for ECM attachment redundant. Our previous study has shown that longer maintenance of spheroids in in vitro cultures resulted in the downregulation of the expression of major integrins [174], consistent with the loss of α6 and β4 integrin subunits in CR spheroids compared to CN spheroids observed in the proteomics study [172]. In addition, ascites is enriched in cytokines like TGFβ and IL-6 capable of inducing mesenchymal features in spheroids [95, 175, 176]. In this context, we have previously described the role of PPL in the induction of EMT in different cancers.

**Fig. 4:**
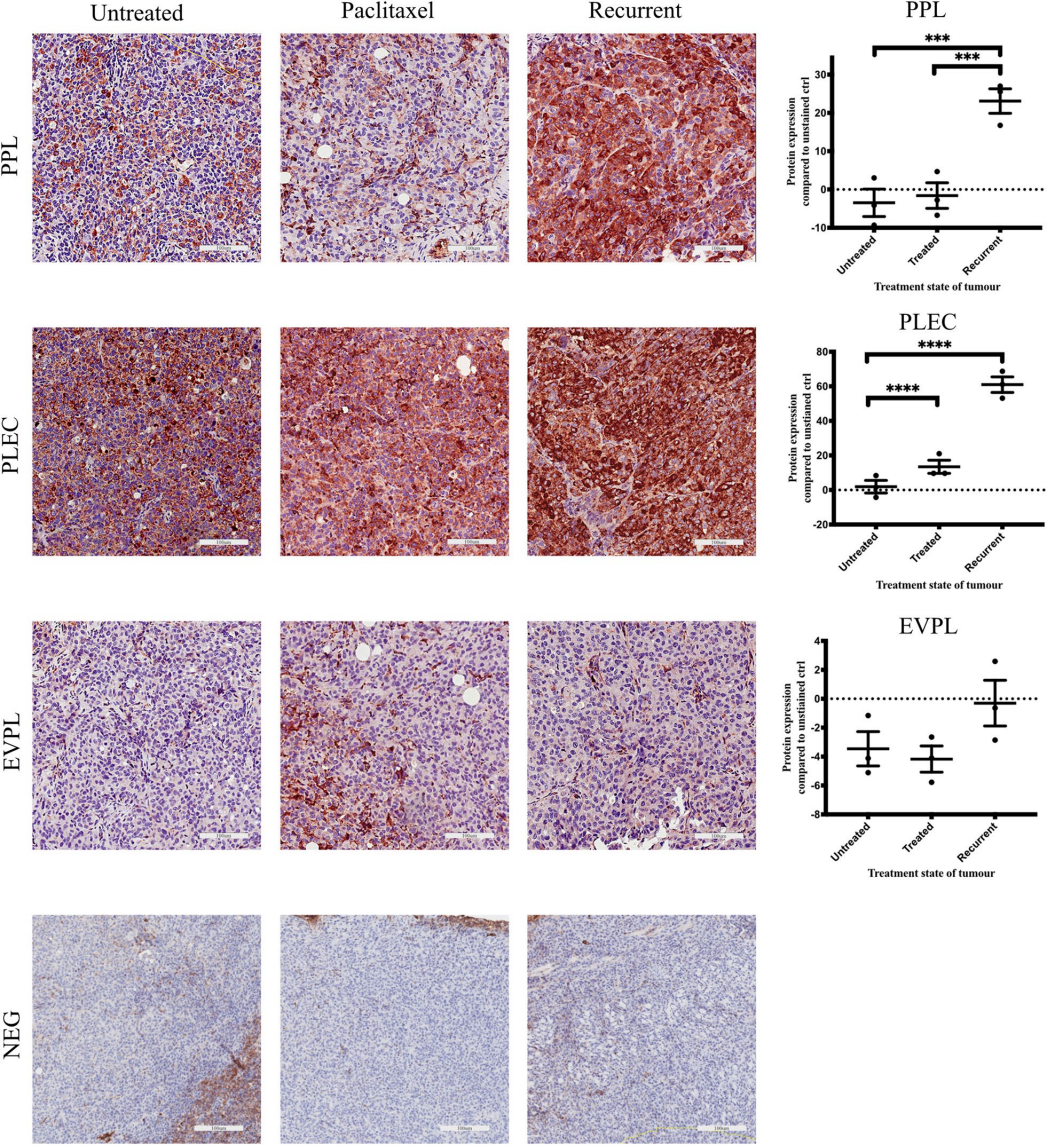
Representative expression of PPL, EVPL and PLEC by immunohistochemistry staining on FFPE tumour xenografts described previously [177]. Animal experiment was performed in accordance with the recommendations in the Guide for the Care and Use of the Laboratory Animals of the National Health and Medical Research Council of Australia. The experimental protocol was approved by the University of Melbourne’s Animal Ethics Committee (Project-1413207.1). Quantification of immunohistochemical staining was performed as described previously [177]. Data is presented as mean ± SEM (n = 3 control mice, n = 3 xenografts from mice treated with paclitaxel, groups 1 and 2). Magnification 20×, scale bar = 100 μm. Significance is indicated by ***p < 0.001; ****p < 0.0001

In this review we present a proof of concept data, Fig. 4, demonstrating that the expression of PPL, PLEC and EVPL was enhanced in a recurrent HEY ovarian cancer cell line derived mouse xenografts described previously [177]. In this experiment, three groups of immune incompetent nude mice were used. Each group was injected intraperitoneally with human HEY ovarian cancer cell line (5 × 10^6^ cells/mouse). The first group was an untreated control, while the second (group 1) and the third group (group 2) of mice received intraperitoneal injection of paclitaxel (15 mg/kg body weight) weekly. Treatment in groups 1 and 2 continued until the endpoint of control untreated mice at which point mice in control and group 1 (paclitaxel-treated) were euthanised. At this point, tumours in groups 1 and 2 reduced to 50% of the size of control tumours. Even though treatment in-group 2 was concluded at the same time, the mice in this group, with 50% reduced tumours compared to control, were kept alive until the experimental end-point (paclitaxel-recurrent). These mice survived 2 weeks longer than control untreated and paclitaxel-treated group 1 mice [177]. Since tumours in group 2 mice, reduced in size on paclitaxel treatment but regained regrowth when they were left untreated, the mice in this group can be treated clinically as a recurrent group. Significant elevation in PPL and PLEC staining in recurrent group 2 compared to group 1 (paclitaxel-treated and culled at the same time as control) and the control group (Fig. 4) was observed by immunohistochemistry, suggesting a potential link between PPL and PLEC expression, paclitaxel resistance and subsequent recurrence in ovarian cancer.

Our previous studies have also shown a sustained loss of PLEC and vimentin in an OCT4A knockdown HEY ovarian cancer cell line [152]. OCT4 is a transcription factor, with the OCT4A variant being required to maintain the self-renewal properties of stem cells and is a nuclear marker of embryonic and CSCs [178]. We have previously shown that stable knockdown of OCT4A in HEY ovarian cancer cells resulted in decreased proliferation, migration and increased chemosensitivity to cisplatin in vitro [179]. Intraperitoneal injection of OCT4A knockdown cells in nude mice significantly reduced the tumour burden with decreased tumour size and invasiveness in peritoneal organs [179]. This resulted in significant elevation in mice survival compared to mice injected with control cells [179]. In a later proteomics study, we identified and validated that stable knockdown of OCT4A in HEY ovarian cancer cell line and the associated xenografts showed a loss of PLEC and vimentin expression [152]. As PLEC is linked to the intermediate filament vimentin through cytoplasmic organelles, and links to nuclear envelope and centrosomes, this result was not unexpected [32]. In the same study, we showed enhanced expression of PLEC and vimentin in ovarian cancer cell lines after treatments with paclitaxel or cisplatin, which was consistent with increased expression of OCT4A in these cells [152]. These findings links novel aspects of plakin regulation connecting key ECM proteins and embryonic transcription factors (OCT4A) in the context of chemoresistance, which is profound in ovarian cancer patients, and the major cause of poor treatment outcomes.

## Conclusions

This review summarises the current understanding about the structure and function of plakins and their roles in normal and diseased (including cancer) biology. Although plakins are important in maintaining the cell–cell, cell desmosome interactions and modulating signalling pathways [2, 11, 48] their role in ovarian cancer remains unexplored. Our observation that plakins are expressed in the epithelial tumour cells of benign, Type I and Type II ovarian tumours but with distinctly different expression patterns suggest a specific role of plakins in ovarian cancer biology. Previously we have shown that plakins (PPL, DSP, PLEC, EVPL) expressed in the ascites-derived tumour cells from patients with chemotherapy-treatment associated recurrence were significantly lower than plakins (PPL, DSP, EVPL, PLEC) in chemonaive ascites-derived tumour cells (Fig. 3). Our current in vivo data in a mouse model indicates significantly enhanced expression of plakins (PPL, PLEC and EVPL) in recurrent mice xenografts that were previously reduced in size with paclitaxel treatment but underwent regrowth after stopping paclitaxel treatments, suggesting an association of plakins with chemoresistance and recurrence in ovarian cancer (Fig. 4).

**Fig. 5:**
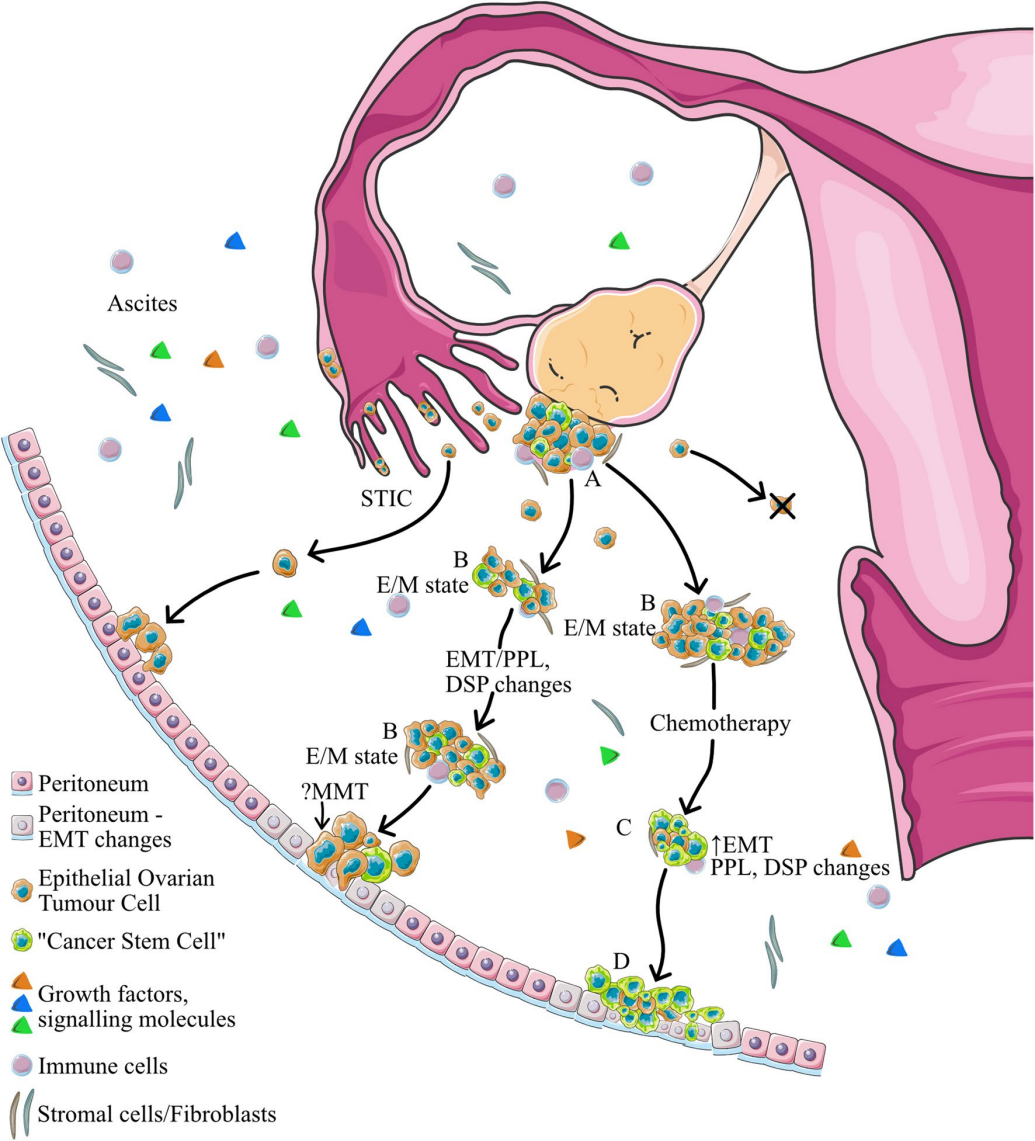
Metastatic dissemination of ovarian cancer requires dynamic and reversible changes of plakin expression in the peritoneal microenvironment as the cancer progresses from primary tumour to floating multicellular spheroids and invasion onto peritoneal lining. The model includes potential involvement of DSP and PPL in STICs, the EMT process at (**A**) primary tumour, (**B**) multicellular aggregate form, (**C**) post-chemotherapy ascites-derived tumour cells and (**D**) tumour invasion of peritoneum and omentum sites where invading tumours trigger MMT and other changes in the surrounding mesothelium

Given the known role of DSP and PPL in EMT, and the complexity of EMT-mediated metastatic process in ovarian cancer, plakin biology is expected to play an important role in each facet of ovarian cancer progression, including progression at the primary site, shedding of tumour cells in the ascites, clustering of tumour cells as spheroids and colonization at metastatic niches. Moreover, detection of PPL and PLEC levels in the serum and ascites of chemonaive and recurrent patients can be undertaken. This may facilitate better understanding of plakin biology in ovarian cancer and may aid in developing a potential role of plakins as biomarkers for early-stage detection as well as monitoring chemoresistance-associated recurrence in patients. Figure 5 describes the potential role of DSP and PPL in different progression phases of ovarian cancer.

## Data Availability

The datasets generated and/or analysed during the current study are not publicly available as it is part of a Ph.D. thesis still under progress. However, if required the data set can be obtained from the corresponding author on reasonable request.

## Abbreviations

EMT: Epithelial-mesenchymal transition
CSC: Cancer stem cells
DSP: Desmoplakin
PLEC: Plectin
EVPL: Envoplakin
PPL: Periplakin
EPPK1: Epiplakin
MACF1: Microtubule-actin cross-linking factor
BPAG1: Bullous pemphigus antigen 1
ABD: Actin binding domain
RACK1: Receptor activated kinase 1
EGF: Epidermal growth factor
EBS: Epidermolysis bullosa simplex
HNSCC: Head and neck squamous cell carcinoma
NKG2D: Natural killer group 2D
CT: Computed tomography
TNBC: Triple negative breast cancer
PDAC: Pancreatic ductal adenocarcinoma
ABC: ATP-binding cassette
CK18: Cytokeratin 18
CDK1: Cyclin dependent kinase 1
CycB: Cyclin B kinase
DSB: Double strand breaks
SCC: Squamous cell carcinoma
NSCLC: Non-small cell lung cancer
PNET: Pancreatic neuroendocrine tumour
TPA: 12-0-Tetradecanoyl phorbol-13 acetate
MCHR1: Melanin concentrating hormone receptor 1
AD: Adenocarcinoma
ASC: Adenosquamous carcinoma
TNT: Tunelling nanotubule
MET: Mesenchymal-epithelial transition
